# Clinical and functional outcomes for bone tunnel vs. suture anchor lesser tuberosity osteotomy repair in total shoulder arthroplasty

**DOI:** 10.1016/j.jsea.2026.100072

**Published:** 2026-08-11

**Authors:** Youssef Galal, Phillip Moroder, Brian C. Werner, Evan Lederman, Anup Shah

**Affiliations:** a Orthopaedic Surgery Department, Loma Linda University, Loma Linda, CA, USA; b Shoulder and Elbow Surgery, Schulthess Clinic Zürich, Zürich, Switzerland; c Division of Sports Medicine, Department of Orthopedic Surgery, University of Virginia, School of Medicine, Charlottesville, VA, USA; d Banner University Medical Group, University of Arizona, College of Medicine, Phoenix, AZ, USA

**Keywords:** Total shoulder arthroplasty, Lesser tuberosity osteotomy, Suture anchors, Range of motion, Patient reported outcomes, ROM, PROs

## Abstract

**Background:**

Subscapularis management with lesser tuberosity osteotomy (LTO) repair in stemless total shoulder arthroplasty (TSA) is particularly different in terms of suture fixation as opposed to stemmed humeral implants. Surgical techniques to achieve optimal soft tissue balance in LTO repairs differ based on the surgeon. The purpose of this study is to determine whether there is a difference in 2 years functional or clinical outcomes in stemless TSA patients treated with LTO repairs conducted with bone tunnels versus suture anchors.

**Methods:**

A retrospective review was performed of a prospectively maintained, multicenter database of primary TSAs performed by multiple surgeons between 2019 and 2023 with minimum 2-year follow-up. A total of 81 patients met the study criteria, including 33 in the suture anchor cohort versus 48 in the bone tunnel cohort. Patient-reported outcomes (PROs) as well as range of motion (ROM) measurements were compared between the 2 groups for baseline results as well as at two year follow-up.

**Results:**

There was no statistically significant difference in age, sex, body mass index, tobacco usage, or diabetes prevalence in either cohort (*P* = .610, *P* = .687, *P* = .980), *P* = .153, *P* = .355, respectively). There was no statistically significant difference in PRO, ROM, or belly press test at baseline between patients treated with suture anchors and those treated with bone tunnels. At two years, there were no statistical differences in outcomes (PROs, ROM, or strength), other than worse forward flexion in the suture anchor group (145° vs. 156°, *P* = .026).

**Conclusion:**

The current study suggests equivalent two year functional and clinical outcomes for patients treated with suture anchors versus the conventional bone tunnels in LTO repair for stemless TSA patients. The 2 groups had similar baseline demographics, ROM, and PRO scores and performed similarly at two years follow-up.

Stemless anatomic total shoulder arthroplasty (aTSA) is growing in popularity in the treatment of glenohumeral osteoarthritis due to decreases in operative time, blood loss, and post-operative pain, as well as preservation of bone and potentially easier revision cases.^13^^,^^14^ In addition, a more anatomic center of rotation found in stemless systems has shown to have improved outcomes compared to stemmed implants.^12^ For all aTSA cases, subscapularis management is a key surgical consideration. As has been well documented, subscapularis repair and healing are critical for optimal outcomes in total shoulder arthroplasty (TSA) and most of the current literature on subscapularis management in aTSA is surrounding the traditional stemmed implant.

Lesser tuberosity osteotomy (LTO) repair and technique in stemmed implants versus stemless implants are inherently different in terms of suture fixation. In stemmed implants, suture placed around the implant provide additional stability through for osseous healing of the lesser tuberosity. Additionally, a stemmed implant can often provide enough fixation early in the post-operative period if the proximal metaphysis has been violated. In contrast, stemless implants do not have these characteristics and therefore place heightened importance in maintaining and preserving the proximal humeral metaphysis which can create challenges for surgeons utilizing a LTO in stemless TSA.

The purpose of this study was to compare suture anchors to traditional drilled bone tunnels during LTO repair in stemless TSA. A comparison of functional and clinical outcomes of patients in either group at 2 years post-operatively was proposed. It was hypothesized that there would be no difference in outcomes between patients who had LTO repairs with either surgical technique.

## Materials and methods

### Study design

A retrospective review was performed of a prospectively maintained, multicenter database of TSAs performed by multiple surgeons between 2019 and 2023. Institutional review board approval and informed consent were obtained prior to the start of the study. Inclusion criteria were primary glenohumeral osteoarthritis, pre-operative patient-reported outcome (PRO) and range of motion (ROM) datapoints, post-operative PRO and ROM datapoints, and a minimum post-operative follow-up of 2 years. Exclusion criteria were revision cases, acute or chronic fractures, avascular necrosis, history of infection, and Walch type C glenoids. A total of 81 patients met the study criteria, including 33 in the suture anchor cohort versus 48 in the bone tunnel cohort.

### Demographics, clinical outcomes, and functional outcomes

The following baseline data were obtained from the database for all patients: age, sex, body mass index (BMI), whether surgery was performed on the dominant arm, tobacco use, and presence of diabetes mellitus. Glenoid morphology was defined by the Walch classification. The following pre-operative and 2-year post-operative outcome scores were recorded and compared: visual analog scale pain score, American Shoulder and Elbow Surgeons score, Western Ontario Osteoarthritis of the Shoulder index score, Single Assessment Numeric Evaluation score, and Veterans RAND 12 mental score. The following ROM data were gathered across 5 different parameters of glenohumeral joint motion in degrees as follows: active forward flexion (FF), active external rotation at the side, active external rotation at 90° of abduction, and active internal rotation at 90°, as well as active internal rotation estimated as the highest spinal level reached. Standardized measurement protocols were implemented across all sites and investigators.

### Surgical technique

Patients were positioned in beach chair position and as standard deltopectoral approach was utilized. Using a large osteotome or sagittal saw, the LTO was performed starting in the biceps groove from lateral to medial, parallel to the insertion of the subscapularis. The anterior humeral head was used as a reference once the rotator interval was incised. After the humeral component has been implanted, subscapularis repair was performed utilizing suture anchors (Eclipse Speedscap, Arthrex, Naples, FL, USA) or bone tunnels through the biceps groove.

### Statistical analysis

Continuous variables (mean age, BMI, PROs, ROM) were analyzed utilizing student *t*-tests. Comparisons of categorical variables (sex, dominant arm, tobacco use, diabetes mellitus, Walch classification, severity of glenohumeral arthritis, subscapularis management, satisfaction) were performed using Chi-squared tests. Student *t*-test and Chi-squared analyses were performed using R (version 4.2.2; R Group, Auckland, New Zealand). A *P* value less than 0.05 was considered significant for all comparisons.

## Results

### Baseline data

Baseline demographics of the 2 cohorts are summarized in Table I. There are 33 patients in the suture anchor group and 48 patients in the bone tunnel group. There was no statistically significant difference in age, sex, BMI, tobacco usage, or diabetes prevalence in either cohort (*P* = .610, *P* = .687, *P* = .980), *P* = .153, *P* = .355, respectively). The majority of patients were classified a Walch A glenoid (n = 56); however, there was no significant difference in Walch classification distribution between the 2 groups.

**Table I:** Demographic comparison of TSA patients in the bone tunnel vs. suture anchor groups.

Demographics of LTO					
Patient characteristics	Anchor (n = 33)		Bone tunnels (n = 48)		*P* value
Demographics					
Age: years (mean, s.d.)	67.2	7.1	66.4	7.3	.610
Sex: female (n, %)	18	55%	24	50%	.687
BMI: kg/m2 (mean, s.d.)	30.5	8.0	30.6	8.4	.980
Dominant arm: yes (n, %)	15	45%	20	42%	.735
Tobacco use: yes (n, %)	3	9%	1	2%	.153
Diabetes mellitus: yes (n, %)	4	12%	3	6%	.355
Walch classification					
A1 (n, %)	25	76%	31	65%	.285
A2 (n, %)	2	6%	2	4%	.699
B1 (n, %)	1	3%	3	6%	.511
B2 (n, %)	5	15%	12	25%	.285
Subscapularis management					
Osteotomy (n, %)	33	100%	48	100%	1.000

*s.d*., standard deviation; *TSA*, total shoulder arthroplasty; *BMI*, body mass index; LTO, lesser tuberosity osteotomy.

Six different demographics were considered for patients in either group including: age, sex, BMI, dominant arm, tobacco use, and whether they had diabetes. The 4 different Walch classification groups are also provided.

Baseline PROs and ROM data are summarized in Table II. There was no statistically significant difference in PRO baseline outcomes between patients treated with suture anchors and those treated with bone tunnels. Additionally, there was no difference in ROM data or belly press strength at baseline for the 2 groups either.

**Table II:** Comparison of baseline PROs and ROM

Comparison of baseline PROs and ROM of LTO patients					
	Anchor (n = 33)		Bone tunnels (n = 48)		*P* value
	Mean	s.d.	Mean	s.d.	
Baseline PROs					
VAS pain	6.4	2.2	5.6	2.3	.113
ASES	37.8	15.0	44.3	15.6	.067
WOOS	36.9	16.3	42.7	15.1	.107
Constant Murley	38.9	15.9	42.4	13.5	.309
VR-12 mental	46.7	13.0	50.8	9.7	.133
Baseline ROM					
Active FF (degrees)	113	28	116	32	.673
Active ER at side (degrees)	37	22	35	20	.660
Active ER at 90 (degrees)	29	32	25	31	.537
Active IR (spinal level)	17	2	18	2	.182
Active IR at 90 (degrees)	16	25	9	18	.164
Baseline strength					
Belly press (lb)	8.1	5.6	8.7	4.4	.111

*s.d*., standard deviation; *PRO*, internal rotation patient-reported outcome; *ROM*, range of motion; *LTO*, lesser tuberosity osteotomy; *VAS*, visual analog scale; *ASES*, American Shoulder and Elbow Surgeons; *WOOS*, Western Ontario Osteoarthritis of the Shoulder; *VR-12*, Veterans RAND 12; *FF*, forward flexion; *ER*, external rotation; *IR*, internal rotation.

This table captures the data for both baseline patient reported outcome data as well as baseline range of motion data pre-operatively. The 5 different baseline patient reported outcome metrics that were utilized include: VAS pain, ASES, WOOS, Constant Murley, and VR-12 Mental. The 5 different baseline range of motion metrics included: active forward flexion, active external rotation at side, active external rotation at 90 degrees, active (based on spinal level), and active internal rotation at 90 degrees (based on degrees). Baseline belly press strength test results were also recorded.

### Clinical outcomes

There were no statistical differences in 2-year functional outcomes (PROs, ROM, or strength), other than worse FF in the suture anchor group (145° vs. 156°, *P* = .026) (Table III).

**Table III:** Comparison of 2-year outcomes.

Comparison of 2-year outcomes of LTO					
	Anchor (n = 33)		Bone tunnels (n = 48)		*P* value
	Mean	s.d.	Mean	s.d.	
2-yr PROs					
VAS pain	0.8	1.9	0.8	1.5	.976
ASES	88.4	17.6	90.9	12.7	.489
WOOS	90.0	17.8	91.1	14.6	.765
Constant Murley	81.7	21.2	79.7	14.9	.647
VR-12 mental	56.0	8.0	54.1	9.7	.328
2-yr ROM					
Active FF (degrees)	145	21	156	18	.026
Active ER at side (degrees)	61	11	65	15	.205
Active ER at 90 (degrees)	66	24	75	20	.096
Active IR (spinal level)	13	3	14	3	.283
Active IR at 90 (degrees)	40	25	34	26	.379
2-yr strength					
Belly press	12.2	5.4	13.1	7.3	.536

*s.d*., standard deviation; *PRO*, patient-reported outcome; *ROM*, range of motion; *LTO*, lesser tuberosity osteotomy; *VAS*, visual analog scale; *ASES*, American Shoulder and Elbow Surgeons; *WOOS*, Western Ontario Osteoarthritis of the Shoulder; *VR-12*, Veterans RAND 12; *FF*, forward flexion; *ER*, external rotation; *IR*, internal rotation.

This table captures the 2-year outcome data post-operatively, through patient reported outcome data as well as range of motion. The 5 different 2-year patient reported outcome metrics that were utilized include: VAS pain, ASES, WOOS, Constant Murley, and VR-12 Mental. The 5 different 2-year range of motion metrics included: active forward flexion, active external rotation at side, active external rotation at 90 degrees, active internal rotation (based on spinal level), and active internal rotation at 90 degrees (based on degrees). Two-year belly press strength test results were also recorded.

The differences between pre-operative and post-operative scores for ROM and PRO were also analyzed as a marker of post-operative improvement (Table IV). No statistically significant differences were observed across either group in PROs, ROM, or belly press strength.

**Table IV:** Comparison of change from pre-op to post-op.

Comparison of change from pre-op to post-op for LTO					
	Anchor (n = 33)		Bone tunnels (n = 48)		*P* value
	Mean	s.d.	Mean	s.d.	
Change in PROs					
VAS pain	−5.6	2.8	−4.8	2.7	.204
ASES	50.6	21.5	46.7	19.9	.407
WOOS	53.7	22.8	48.0	19.9	.255
Constant Murley	42.8	25.9	39.9	17.7	.606
VR-12 mental	8.2	13.7	2.9	8.9	.060
Change in ROM					
Active FF (degrees)	31	36	42	31	.207
Active ER at side (degrees)	23	21	31	22	.177
Active ER at 90 (degrees)	38	35	52	42	.133
Active IR (spinal level)	−4	4	4	4	.958
Active IR at 90 (degrees)	22	34	23	27	.924
Change in strength					
Belly press	3.0	5.7	3.7	7.1	.662

*Pre-op*, pre-operative; *Post-op*, post-operative; *s.d.*, standard deviation; *PRO*, patient-reported outcome; *ROM*, range of motion; *LTO*, lesser tuberosity osteotomy; *VAS*, visual analog scale; *ASES*, American Shoulder and Elbow Surgeons; *WOOS*, Western Ontario Osteoarthritis of the Shoulder; *VR-12*, Veterans RAND 12; *FF*, forward flexion; *ER*, external rotation; *IR*, internal rotation.

This table captures the change between baseline and post-operative outcomes for both baseline patient reported outcome data as well as baseline range of motion data. The 5 different baseline patient reported outcome metrics that were utilized include: VAS pain, ASES, WOOS, Constant Murley, and VR-12 Mental. The 5 different baseline range of motion metrics included: active forward flexion, active external rotation at side, active external rotation at 90 degrees, active internal rotation (based on spinal level), and active internal rotation at 90 degrees (based on degrees). The difference between 2-year and baseline belly press strength test data was also recorded.

## Discussion

The current study finds FF to be significantly worse in the suture anchor group and otherwise equivalent functional and clinical outcomes with a suture anchor and bone tunnel repair for LTO in stemless aTSA at two years post-operatively. The decreased FF at two years in the suture anchor group was 11°. This finding warrants future studies with longer follow-ups needed to determine whether this difference persists, resolves, or worsens over time. Biomechanical testing studies have demonstrated the failure mode and strength of suture anchors, primarily in soft tissue to bone healing.^3^ However, these studies have been primarily been in open subscapularis repairs or in standard stemmed aTSA. Soft, all suture anchors also permit smaller drill holes and increased subscapularis coverage on the lesser tuberosity, which may be leading to greater utilization.^15^^,^^16^

Stemless TSA has been gaining popularity due to humeral bone preservation and decreasing operative time and blood loss.^13^^,^^14^ Meta-analysis of current prospective randomized controlled trials comparing use of stemless to stemmed humeral components in TSA, have shown little difference in post-operative functional outcomes or revision rate.^9^ Subscapularis management has additional considerations in stemless TSA due to a more anatomic center of rotation and less points of fixation then in a stemmed humeral component.^12^ LTO is a common technique for subscapularis management in the setting of TSA. In LTO repair, suture holes are drilled near the osteotomy site and suture is either passed through bone tunnels or secured through suture anchors before being passed through the subscapularis tendon medial to the osteotomy fragment.^11^ This allows for access to the glenohumeral joint through retraction of the attached subscapularis without disrupting any muscle like in subscapularis peel and tenotomy. After implantation of the final TSA system the lesser tuberosity is resecured to the proximal humerus with heavy non-absorbable suture, permitting bone-to-bone compression healing. However, some surgeons opt to not use LTO in stemless TSA cases due to concerns about violating proximal humeral bone.^10^ In stemless implants there is commonly more difficulty achieving robust fixation, so bone stock preservation is an essential consideration. The major concern with LTO is that the osteotomy may extend to the metaphyseal-calcar region and theoretically disrupt the humeral bone stock that is relied upon for the stemless implant fixation.

The proposed benefits of LTO as a technique is the direct osseous healing which can be tracked radiographically, paired with some studies demonstrating an improvement in functional testing for subscapularis immediately post-operatively.^5^^,^^6^ Additionally, some studies have reported less stress shielding in stemless TSA patients that received an LTO as opposed to other subscapularis management methods such as peel and tenotomy.^1^ In stemmed TSA, there are multiple biomechanical and randomized control trials analyzing different subscapularis repairs which show no significant clinical difference in post-operative outcome or repair strength between various repair techniques.^2^^,^^4^^,^^7^^,^^8^ A recent randomized control trial by Lapner et al^7^ reported no difference regarding primary outcome of subscapularis strength at 24 months between peel and LTO groups for stemmed TSA patients. There has not been the same depth of literature in stemless TSA despite the differences in soft tissue balance inherent to the prosthetic's design.

This study features multiple limitations inherent to the study design. As a retrospective cohort review there are confounding variables that may potentially affect each group's functional or clinical performance that the study may have not accounted for. However, demographic data as present in (Table I) demonstrate no statistically significant differences in patient characteristics between the 2 groups for the factors that were analyzed. Another limitation is the relatively small population of patients. An increased sample size for both cohorts would allow for improved power. Future studies regarding this topic should focus on surgical technique literature on suture anchor placement for LTOs in stemless TSA. Techniques for subscapularis repair are different in stemless TSA. Despite this, there remains little literature on the nuances of rotator cuff and soft tissue management in stemless TSA implants. This warrants the need for additional studies focusing on surgical technique as well as biomechanical testing of various constructs.

## Conclusion

The current study suggests equivalent two-year functional and clinical outcomes for patients treated with suture anchors versus the conventional bone tunnels in LTO repair for stemless TSA patients. The 2 groups had similar baseline demographics, ROM, and PRO scores and performed similarly at two years follow-up. This may suggest that the use of suture anchors may be equivalent in effectiveness to bone tunnels; however, additional biomechanical data would likely be most useful in determining tensile properties of each repair.

## Disclaimers:

Funding: This study is funded by Arthrex, Inc. Grant no.: AIRR-00608-56.

Conflicts of interest: Brian C. Werner is a consultant for Arthrex and receives research support from Biomet and Flexion Therapeutics.

Anup Shah and Evan Lederman are consultants for and receive royalties from Arthrex, Inc.

Phillip Moroder is a consultant for and receives royalties from Arthrex, Inc. and Medacta.

Any additional authors, their immediate families, and any research foundations with which they are affiliated have not received any financial payments or other benefits from any commercial entity related to the subject of this article.
